# Evaluation of the Production Performance and the Meat Quality of Chickens Reared in Organic System. As Affected by the Inclusion of *Calliphora* sp. in the Diet

**DOI:** 10.3390/ani11020324

**Published:** 2021-01-28

**Authors:** Ainhoa Sarmiento-García, Carlos Palacios, Inmaculada González-Martín, Isabel Revilla

**Affiliations:** 1 Area of Animal Production, University of Salamanca, 37007 Salamanca, Spain; cpalacios@usal.es; 2 Department of Analytical Chemistry, Nutrition and Bromathology, University of Salamanca, 37008 Salamanca, Spain; inmaglez@usal.es; 3 Area of Food Technology, Polytechnical High School of Zamora, University of Salamanca, 49022 Zamora, Spain; irevilla@usal.es

**Keywords:** alternative protein source, *Calliphora* sp., insects, organic poultry, performance, meat quality, larva meal

## Abstract

**Simple Summary:**

Currently, organic systems face the problem of the low availability of protein for feeding monogastric animals. Insects are part of the diet of free-range animals, and can be a possible substitute for other protein sources such as soybean meal. In the EU the production of insects is not eligible in organic production due to the absence of standards for these products. Therefore, more studies are needed to support the inclusion of insects in animal feeding. This study aimed to evaluate the inclusion of *Calliphora* sp. (CLM) in the first phase of chicken growth in organic production systems. Similarly, the quality of the meat of these chickens was analyzed and near infrared spectroscopy (NIRS) was used as a tool to classify the meat according to the diet they had consumed. This study showed that the use of CLM in feeding of organic chickens would be a good alternative, thus obtaining chickens with similar meat quality and productive developments, in contrast to those chickens that had been fed traditional proteins. In addition, the NIRS could be useful for the classification of meat from chickens based on the diet they had received.

**Abstract:**

The use of insects can be a possible source of protein. This study uses *Calliphora* sp. larvae (CLM) as a protein source in 320 one-day-old medium-growing male chicks (RedBro) during their first month of life. Chickens were randomly assigned to four dietary treatments. Each group consisted of 10 animals, and a total of 8 replicas. Control group was fed with a certified organic feed. The experimental treatments were supplemented with 5% (T2), 10% (T3), or 15% (T4) of CLM, reducing in each case the corresponding percentage of feed quantity. Productive development and meat quality were analyzed, and near infrared spectroscopy (NIRS) was used as a tool for classifying the samples. Chickens of T4 showed greater final body weight and total average daily gain, but they reduced consumption and feed conversion ratio (FCR). The chicken breast meat of T4 had lower cooking losses and higher palmitoleic acid content (*p* < 0.01). NIRS classified correct 92.4% of samples according to the food received. CLM is presented as a potential ingredient for the diet of medium-slow growing chickens raised in organic systems.

## 1. Introduction

The lack of availability of protein raw materials for feeding organic animals, especially pigs and poultry, is one of the main limitations encountered by this sector. The productive development, health status and welfare of the animals depend directly on the adequate supply of protein in the diet [1]. Therefore, the search for protein alternatives that are sufficient both in quality and quantity is a priority for these producers.

Insects are a part of the natural diet of free-range fish and monogastric animals throughout the world [2,3]. Insects are one of the options which are being considered as an alternative to traditional protein sources (e.g., soybean meal), due to their high quality and quantity of their protein [2,3]. Insects contain between 30% and 70% of protein on a dry matter basis [4] and have a high nutritive value being also rich in fats, minerals and vitamins [3] In addition, the low competitiveness with human food [5,6] and the reduction in the environmental impact in terms of energy cost and use of arable area [7] make insects a promising food source in a green production environment. However, currently the use of insects in bird feeding is currently not allowed in the European Community. Given the potential ecological advantages associated with a good acceptance between producers and consumers [8], it seems likely that political legal frameworks may change in the near future, making it possible to use insect proteins in bird feeding; being a valuable potential also for organic farming [9].

The initial period (30 days) is considered the most important one in broiler production, as growth and development occur at a very strong rate in this phase. In fact, in this first phase, the weight of the chicken increases the initial weight up to four times. This fact determines its subsequent growth [5,10]. That is why the contribution of nutrients of high biological and nutritional quality, such as insects, are key to the correct productive development of the chickens. Therefore, the inclusion of insects as a protein source for the feeding of chickens, is presented as an opportunity for organic poultry. However, further research is required to determine the benefits of using insects in chicken feeding.

Currently, there are significant knowledge gaps in the field of insect production, particularly in Europe, where insects are not considered a traditional food [4,11,12]. Although from the point of view of the nutrient composition of the insects, they could be suitable for the nutrition of the birds [4,6,7,9], other aspects must be worked on, including reducing the costs of insect production [7]. On the other hand, it is well known that, in monogastric animals, the composition and characteristics of the meat are strongly influenced by the food consumed. Therefore, the quality of the meat of animals that have consumed insects in the diet should be studied [13].

During the past years, many consumers have been interested in the non-compositional aspects of meat related to quality, as well as the intrinsic characteristics of the animals (species, breeds), the geographical origin, the food received, the productive management or the post-mortem strategies [14]. Thus, the demand for proper analytical methods for authenticity assessment of food products is currently increasing dramatically, representing one of the major concerns for different stakeholders. Strict controls both throughout the production and during the marketing process are required. In this sense, Near Infrared Spectroscopy (NIRS) is presented as a powerful tool. The basic idea of the application of NIR technology to solve authentication problems is based on the generation of the spectral footprint of food [15].

Based on the foregoing, the present work aims to evaluate the effect of the inclusion of *Calliphora* sp. (CLM) on the productive performance of medium-slow-growing chickens raised in organic production systems during their first month of life, indeed physico-chemical quality of the meat of chickens that received different percentages of a supplement of CLM. Likewise, the ability of the NIRS to properly classify the carcass samples was performed, based on the percentage of inclusion of CLM was evaluated.

## 2. Material and Methods

### 2.1. Ethical Statement

This study was carried out with farm animals, which due to their characteristics did not require a special certification according to the method of breeding laboratory animals. Nevertheless, these procedures were authorized by the Animal Experimentation Service (SEA) of the University of Salamanca, in line with the standards set by the Confederation of Scientific Societies of Spain (COSCE) (Project Identification Code IDE2019/041). Animals were reared in accordance with the Spanish Policy for Animal Protection [16], which meets the European Union Directive [17] on the protection of animals, and on the animal welfare of farm animal regulations [18], which establishes the minimum standards for the protection of chickens destined for meat production.

### 2.2. Animals and Husbandry

A total of 320 1-day-old medium-growth male chicks (*Gallus gallus domesticus*) RedBro lineage were selected for the study. This is an intermediate-slow-growing hybrid named REDJA Ki, which comes from a cross between the male RedBro and the female JA57Ki (Hubbard^®^). Chicks were vaccinated against Newcastle disease and Infectious Bronchitis, and they were randomly allotted to the four dietary treatments (8 pens/treatment and 10 birds/pen). The period of inclusion of the treatment comprised until day 28 life of the animals.

Chicks were randomly allocated in 4 independent areas within the same facility, sharing identical climatic conditions. Each of the areas was divided into 8 divisions (replicas), and each replica had 10 chickens. Each pen (1.20 m wide × 1.20 m long) used barley straw as a bed and had not access to the outside. Each pen was provided with individual feeder and drinker to allow *ad libitum* intake. The temperature inside was maintained at 35 °C the first week, 30 °C the second, and 25 °C the third and fourth weeks with natural lighting.

On day 30 of life, randomly selected five chicks per group were taken to a certified slaughterhouse (n = 160) where they were slaughtered in accordance with the regulations for organic production animals [19]. The chickens were weighed after slaughter to determine the slaughter weight. After 15 min post-mortem, the front quarters were removed, weighed, and sent refrigerated to the laboratory. At the lab, the front quarters were deboned to obtain the breast muscle (m. *Pectoralis major*). The breast skin was removed and the chicken breast meat were weighed and frozen at −18 °C.

### 2.3. Diets

The basis of the diet for all groups was a certified organic compound feed, made by an authorized feed factory (Coslada, Madrid, Spain). This feed fully complies with the requirements for the production of chicken slow-medium growing [20]. Control group only had the certified compound feed as a food source, for the experimental treatments it was supplemented with 5% (T2), 10% (T3), 15% (T4) of CLM reducing in each case the corresponding percentage of feed quantity. *Calliphora* sp. was obtained from a commercial source (Zamora, Spain). The objective of reducing the feed for the inclusion of the percentages of CLM is due to the proposition that it is the farmer himself who could carry out this feed in the future on his farm; considering CLM as one more raw material.

Chemical composition of CLM and experimental feeds were analyzed using standard procedures (AOAC, 1995): water content by drying at 105 °C; protein content by the Kjeldahl method; fat content by Soxhlet extraction; and ash content by incineration. Fatty acid profile of CLM and the different experimental diets (T1, T2, T3, T4) were analyzed. Fatty acid profile was determined by gas chromatography according to the method described by Lurueña-Martinez et al. [21]. The amino acid content was analyzed for the experimental treatments, the samples were pretreated by acid hydrolysis with hydrochloric acid and subsequent separation and quantification of the amino acids by liquid chromatography (LC-MS) was carried out. The composition of CLM is given in Table 1. The basal diet, whose composition is given in Table 2. Table 3 shows the fatty acid profile for each of the experimental diets. As it is observed the diets were not isoprotein or isoenergetic.

### 2.4. Growth Performance, Weight Gain, Feed Intake and Feed Conversion Ratio

Feed intake (FI), deaths of the animals and their general condition were registered in each pen.

The animals were weighed at a regular interval of one week. (W1, W2, W3 W4, W5, respectively) with a precision weighing balance (±0.01 g). The average daily gain (ADG) was calculated as the difference between 2 consecutive body weight (W) measurements, divided by the number of days between weighing. Total ADG is the average daily gain between W1 and W5 corresponding to the total period of the experiment. Feed conversion ratio (FCR), were calculated by using the documented data of total ADG and FI.

FCR = FI/ADG

### 2.5. Meat Characteristics Analysis

Prior of the analysis, breasts were defrosted at 4 °C for 24 h. The pH measurement was taken from the pectoral muscle with a CRISON pH METER BASIC 20^®^ (Hach Lange Spain, L’Hospitalet de Llobregat, Barcelona, Spain) equipped with a penetration electrode. The color determination of the meat samples was carried out using a colorimeter HunterLab MiniScan model XE Plus (Hunterlab, Virgina, EEUU) equipped with a 25 mm measuring head and diffuse/8° optical geometry. The meat color was measured on the outer side of the pectoral muscle without skin after 1 h of exposure to the air, determining the parameters L* a* b* using an observer of 10° and the illuminant D65 in the CIELab space. Expressible juice was measured according to a modification of the method of Grau et al. [22] as described by Pla et al. [23].

Subsequently, the chicken breast meat was divided longitudinally into two subsamples (A1, A2). A1 samples were chopped up and homogenized. Moisture, ash, and fat were determined by AOAC-approved methods (AOAC, 1990). Fat and ash were reported as a percentage of dry matter (DM). Thiobarbituric acid reactive substances (TBARS) assay was performed as described by Buege and Aust [24]. Samples (5 g) were mixed with 1 mL of water with an UltraTurrax (IKA, Staufen, Germany). 1ml of the extract was mixed with 50 μL of butylated hidroxianisole (7.2%) and 2 mL of 0.375% TBA-15% TCA solution. The mixture was heated for 30 min in a boiling water bath (95–100 °C) to develop a pink color, cooled with tap water, centrifuged at 6500 rpm for 5 min (Sigma 4k15), and the supernatant was measured spectrophotometrically at 532 nm using a UV 1280 spectrophotometer (Shimadzu Co., Kyoto, Japan). TBARS were calculated from a standard curve of malondialdehyde, a breakdown product of tetraethoxypropane (TEP) used in preparation of the standard curve. TBA number was calculated as mg MDA/kg sample. All the analyses were performed in triplicate.

Intramuscular lipids were extracted according to the method described by Folch et al. [25] method. (1957). Five grams of sample were mixed with 15 mL of chloroform/methanol (2:1 *v/v*). The mixture was homogeneized with an UltraTurrax ((IKA, Staufen, Germany) and filtered. The extraction was repeated three times. Subsequently KCl (0.88%) was added to the filtered liquid to achieve a final ratio of 4:1. The obtained mixture was shaken vigorously and the byphasic system was centrifugated at 3500 rpm for 5 min (Sigma 4K15, Osterode am Harz, Germany). The aquous phase was discarded and 10 mL of water and 10 mL of methanol was added and the mixture was centrifugated at 3500 rpm for 5 min. Afterwards, the organic layer was separated and evaporated under vacuum in a rotavapor (Büchi R-200, Flawil, Switzerland) until dryness. The fatty acid composition of lipids was determined according to the method described by Lurueña-Martínez et al. [21]. Extracted fatty acids were methylated with KOH 0.2 M in anhydrous methanol and then analysed by gas chromatography (GC 6890 N, Agilent Technologies, Santa Clara, CA, USA) using a 100 m × 0.25 mm × 0.20 µm fused silica capillary column (SP-2560, Supelco, Inc, Bellefonte, PA, USA). One µL was injected into the chromatograph, which was equipped with a split/splitless injector and a flame ionization detector (FID). The oven temperature programme was started at 150 °C followed by increases of 1.50 °C/min up to 225 °C, at which point it was maintained for 15 min. The temperature of the injector and detector was 250 °C. The carrier gas was helium at 1 mL/min and the split ratio was 20:1. The different fatty acids were identified by the retention time using a mixture of fatty acid standards (47885-U Supelco, Sigma-Aldrich, Germany). The fatty acid contents were calculated using chromatogram peak areas and were expressed as g per 100 g of total fatty acid methyl esters. Fatty acids were identified by comparing the FAME retention time with the standard Supelco 37 component FAME mix (Supelco, Bellefonte, PA, USA). The fatty acids concentrations were expressed as g/100 g of fat. Moreover, the ratios n-6/n-3 and PUFA/SFA were calculated. The atherogenic index (AI = [C12:0 + (4 × C14:0) + C16:0]/(PUFA + MUFA)) and the thrombogenic index (TI = (C12:0 + C14:0 + C16:0)/[(0.5 × n6) + (3 × n3) + (0.5 × MUFA) + (n3: n6)]) were also calculated according to Ulbricht and Southgate [26].

The NIR spectra was recorded on subsample A2, that were thawed at 4 °C for 24 h and subsequently the recording was carried out. A Foss NIRSystem 5000 (Foss Iberia SA, Barcelona, Spain) with a standard 1.5 m 210/210 bundle fiber-optic probe, (Ref. nº R6539-A) was used for NIR spectroscopy. The probe used a remote reflectance system and a ceramic plate as a reference. The window was made of quartz with a 5 cm × 5 cm surface area. The remote reflectance fiber-optic probe was directly applied to the meat samples without any preparation. The spectral range was set at 1100–2000 nm and the spectra were recorded at 2 nm intervals and 32 scans were taken for both the reference and the samples. All samples were analyzed in triplicate in order to minimize sampling error.

Later, A2 samples were weighed and packaged in plastic bags and heated to 75 °C (center piece) and then cooled. The internal temperature of the steaks was measured with a ChecktemW1 digital thermometer (Hanna Instruments, Eibar, Spain). After being cooked, the chicken breast meat was weighed to determine the cooking loss. When the temperature of the center of the samples had reached 75 °C, the samples were cooled under running tap water until the temperature had fallen to 15 °C, removed from the bags, and weighed. The cooking loss was determined as the difference in weight between raw steaks and cooked steaks divided by the weight of the raw sample and multiplied by 100 to express it as a percentage. This analysis was performed in duplicate. The Instrumental Hardness (Warner Braztler Shear Force) was determined from the cooked samples. Three portions of the 1 × 1 section of 3 cm in length were prepared and analyzed using the Texture Analyzer TAXT2i (Stable Micro Systems, Surrey, UK) equipped with a Warner-Bratzler blade. A cut was made perpendicular to the direction of the muscle fiber at a speed of 1 mm/s; the maximum shear force (WBSF) was registered.

### 2.6. Statistical Analysis

#### 2.6.1. Growth Performance, Weight Gain, Feed Intake and Feed Conversion Ratio

The significance of the effect of CLM flour in the feed was obtained by using the general linear model procedure (GLM). The diet was used as a fixed factor, while the weight gained weekly, ADG and FCR were used as the dependent factors. Means and standard deviations were calculated for all variables. In addition, the coefficient of variation between (CV) the different treatments and weeks was carried out, in order to verify the degree of uniformity per group. A Student’s *t* test was also carried out to check the growth of each replica over the weeks.

The significance level at which differences were considered was *p* < 0.05. Values between 0.05 < *p* < 0.10 was considered a trend. All statistical analyses were carried out using the SPSS Package 23 (IBM SPSS Statistic, 2017).

#### 2.6.2. Meat Chemical Composition and NIRS

The significance of the effect on meat quality was obtained by using GLM. Means and standard deviations were calculated for all variables. The significance level at which differences were considered was *p* <0.05. Values between 0.05 < *p* < 0.10 was considered a trend. All statistical analyses were carried out using the SPSS Package 23 (IBM SPSS Statistic, 2017).

The residual mean squares method (RMS-X residuals) was carried out with the Win ISI 1.50 (Infrasoft International, State College, PA, USA) software using the whole NIR spectrum. Different combinations of the following mathematical treatments (none, multiplicative scatter correction (MSC), standard normal variate (SNV), detrend (DT) or SNV-DT), first or second derivatives, and several gaps over the derivative were calculated, and different numbers of data points in a running average and one or two smoothing were assayed and coded as follows (None 2,4,4,1) as previously described by González-Martín et al. [27]. The best mathematical treatment for distinguishing between the samples was selected taking into account the highest percentage of correctly classified samples.

## 3. Results

### 3.1. Growth Performance

No mortality or illness symptoms were observed during the entire trial among all the treatments, and the survival rate was 100% in the whole experimental period. There was a significant increase in weight for each of the weights performed according to Student’s t (*p* < 0.001).

Table 4 shows growth performance for chickens from different diet treatment. The initial weight (W1) of the chicks did not show significant differences between the four treatments. In W2 and W3, even though there were groups with significantly higher weights, there was no proportional or related behavior with the diet provided. Although it is true that from the W4 weighing it was found that the inclusion of CLM significantly increased the weight (*p* < 0.005) as the concentration of CLM in the feed increased. At the end of the first chick growth phase (W5) the animals that had received the 10% and 15% treatment had a significantly higher weight (*p* < 0.001) compared to 5%.

Regarding the ADG (Table 4), it is observed that there are statistically significant differences (*p* < 0.01) due to the inclusion of CLM for all the intervals considered. The chickens that had received 15% larval meal had a higher ADGt than the T2 group, which was the group that presented the lowest value. As was the case for weight, ADG 5-4 showed two clearly differentiated groups, in which the increase in CLM concentration (T3 and T4) showed higher values (*p* < 0.05) compared to the control (T1) and the group with less inclusion of CLM (T2).

For total feed intake (FI), control group (T1) consumed a greater amount (*p* < 0.001) of feed with respect to the groups that had received CLM (Table 4), group T2 and T4 had a lower FI than group T3. However, it should be noted that there were no rejections in the consumption of the feed during the duration of the experiment, which is justified by the higher ADG and weight found in these groups (T3 and T4).

Our study showed differences in the FCR (*p* < 0.0001); as the concentration of CLM in the feed increased, the value of the FCR decreased (Table 4). The groups that had consumed a greater amount of CLM (T3 and T4) had a lower FCR.

Taking into account the consumption of these nutrients per animal (g), it was observed that the chickens belonging to the T4 and T3 group ingested a greater amount of protein and digestible energy for all study period. On the other hand, the chickens in the control group (T1) consumed a higher amount of arginine and lysine compared to the groups that had included CLM in the diet for the total study period (T2, T3, T4). (Table 4).

CV was between 7.8 and 15.8%; for the total study period in all dietary treatments. The smallest CV value was for group T2 at the first weighing (W1); while the highest CV value was for T3 at the second weighing (W2). An increase in CV was observed in group T2 and T3 for the second weighing (W2) (12.59; 15.80%); while in the fourth weighing (W4) the increase in CV was evident in all treatment groups (T1 10.54; T2 10.65; T3 10.64; T4 11.55). It is important to mention that in the last weighing (W5) the value decreased for all groups. This indicates that all treatments groups had a homogeneity close to 90% in W5.

### 3.2. Meat Quality

Although the 15% treatment chicks (T4) had a higher slaughter weight (W5) (*p* < 0.001) than the rest of the groups; our study did not show differences neither for the weight of the front quarter nor for the breast meat (Table 5). The chemical composition of the breasts was studied in terms of ash moisture, fat and fat oxidability (TBARS) (Table 5). The chemical composition of the breast did not differ between the four experimental groups, thus there were not significant differences of moisture, protein, lipid and ash content.

The lipid profile of the meat from different treatments was analyzed, as well as the most representative lipid fractions (Table 6). For all dietary treatments, predominant fatty acids in chicken breast meat was palmitic acid (C16:0) as SFA; oleic acid (C18:1 n-9c) as MUFA and linoleic acid (C18:2 n-6c) as PUFA. Oleic and palmitic acids were the most abundant fatty acids in the analyzed samples. On the other hand, among the SFA the fatty acid that was found in the smallest amount was margaric acid (C17:0), while of the MUFA it was myristoleic acid (C14:1 n-5), and in the PUFA it was eicosadienoic acid (C20:2 n-6). The individual composition of the fatty acids did not show significant differences between the different treatments used for most of the fatty acids analyzed. Differences (*p* < 0.001) were only observed for palmitoleic acid (C16:1 n-9), for which an increase in its concentration was observed as the replacement of the feed by CLM was increased.

Regarding the fractions of fatty acids, differences were observed (*p* < 0.05) for the content of SFA (Table 6). The values of control treatment (T1) and of the group with the highest inclusion of CLM (T4) were intermediate to the previous ones. Despite not finding differences for the content of MUFA, a trend (*p* = 0.055) was observed towards the animals belonging to the groups with the highest inclusion of CLM (T3 and T4) having a higher concentration of these acids compared to the groups T1 and T2. Regarding TI, T2 group shows an increase (*p* < 0.05) of this ratio; while the AI did not show differences between diets.

### 3.3. Technological Characteristics

Technological characteristics, in terms of color, pH, expressible juice. cooking losses and instrumental texture of chicken breast meat depending on the diet received are shown in Table 7. No differences were found for color (L*, a*, b*), pH, expressible juice and texture for meat from different experimental diets. On the other hand, T4 chickens showed a lower value of cooking losses (*p* < 0.001) in the chicken breast meat.

### 3.4. Discriminant Analysis Using NIR Spectra

**Figure 1:**
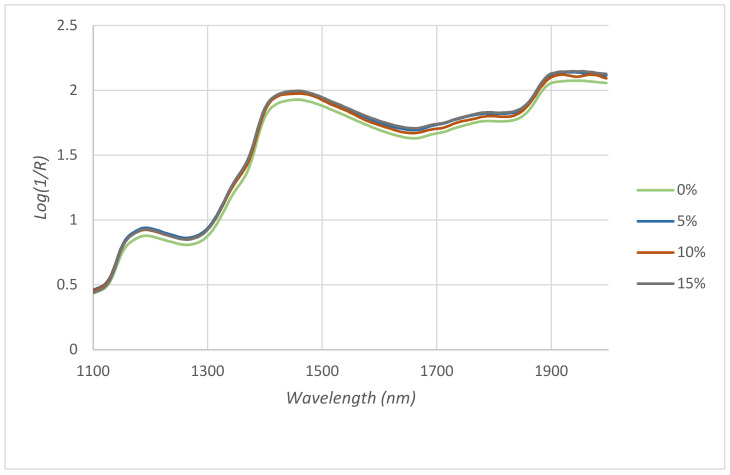
Spectra from NIRS registry of chicken breast meat as a function of the inclusion of CLM in the chicken diet.

The discriminant analysis of the whole NIR spectra of the 160 samples (40 samples from each of the different feeding groups T1, T2, T3 and T4) was carried out using the RMS-X residuals method. This analysis implies the pre-treatment of the spectra with different combinations of mathematical treatments (MSC, SNV, DT or SNV-DT), derivatives, and smoothing procedures. The optimal treatment is that giving the highest percentage of correctly classified samples. In this case, SNV 2,4,4,1 allowed correct classification of 100% of the control samples (T1), 85.71% of the samples of the 5% group (T2), 88.8% of the 10% group (T3) and 91.66% of the 15% group (T4). The average spectra of the four different groups are shown in the Figure 1. It is observed that the line corresponding to the samples from the control chickens (T1) is the one that can be differentiated almost entirely from the rest, which confirms the high capacity of the system classify these samples correctly (100%).

## 4. Discussion

This study provides new insights into the inclusion of insects in the diet of medium-slow growing chickens reared in free range conditions. The present work is the first carried out using *Calliphora* sp. as part of the feeding in medium-slow growing chickens in organic systems. There is only one research on meat quality to evaluate the inclusion of insects as a meal for chickens raised under organic systems and fed with *Tenebrio molitor* (TM) as an insect [28]. The rest of the studies shown below have been carried out in industrial production (broiler).

The best results in the final weight (W5) were in the animals that had consumed a major amount of CLM, which may be related to the greater amount of protein and energy consumed by these animals. This is observed in Table 4, where the consumption per animal and study period of these nutrients is shown. These results are in line with the finding of Teguia et al. [29] who shows a lower weight from control group, while the chickens fed with the maggot meal diet obtained the highest weight. Although the studies are not completely comparable due to the different insects used, this study suggests that the use of insects could improve chickens’ weights at 30 days, to those that received diets containing traditional feeding ingredients.

The inclusion of CLM in chicken diets improve the chicken growth performance in terms of ADG. These results are in agreement with the literature [24]. These authors found a significant improvement in ADG in chickens that had received insect meal compared to the control during the first period of life (1–30 days). In contrast, other authors [30,31,32] did not found variations on ADG when insect meal was included in the diet of the animals, in terms of control. When the level of flour inclusion was considered, no significant differences were found between the total ADG of groups T3 and T4. These findings suggest that there is no proportionality between the degree of inclusion of CLM and the final weight or the ADG during the first month of life.

An increase in the FI of chickens that had included CLM in the feeding was shown. This agrees with the findings of Lachicha et al. [33], who considers that when insects are offered as part of the bird’s diet, a very high intake occurs in the first weeks of life; but it stabilizes from the fourth week onwards. This indicates that when birds are in their natural habitat, they incorporate insects into their diet. Ballitoc and Sun [34] justified an increase in the consumption of the chickens that received TM attributing it to the increase in palatability of these mixtures and relating it to the natural behavior of these animals when they are free. However, other authors [35,36] consider that the inclusion of more than 25% of fly larvae in the diet of chickens can cause a decrease in consumption as a consequence of darkening. It has been suggested that the optimal inclusion rate to avoid rejections depends on the age of the animals. Awoniyi et al. [37] found better results when partially replacing fishmeal (25%) with worm meal, than when total replacement occurred in old broilers; while young broilers did not reduce their consumption when offered feed that had a total replacement of the protein source.

The FCR was improved in the chickens that had eaten a higher amount of CLM (T4). This can be related to the better protein quality of the CLM larva included in the feed (Table 1). The chicks that received treatment T3 and T4 consumed a higher amount of protein compared to the control, which is responsible for the decrease of FCR and the increase in weight (Table 4). Similar results were found by Ballitoc and Sun [34], who showed a decreasing trend in FCR values of broiler fed on TM from 0% to 10% of inclusion in the diet. Different authors [7,33,38,39] have described improvements over FCR in those animals that received insects as a source of protein in their diet. These authors attributed the best value for the FCR to the better protein quality of the larvae compared to other raw materials. On the other hand, Dabbou et al. [40] observed an improvement FCR during the first period of life of the animals, but the growth period to slaughter (10–35 days) had a worse FCR value during the first period.

This study showed a high degree of homogeneity for the slaughter weight of chickens (W5) measured by CV, and the values obtained are shown in the results section. Group uniformity can be expressed as the coefficient of variation in alive weight, increased CV values are synonymous with decreased of uniformity. The level of uniformity basically dictates the final result; high CV values indicate growth retardation, rejections, and poor FCR [41]. The high degree of homogeneity observed in the group is justified by the high-quality diet received (in terms of the amount of protein ingested). This agrees with what has been published by Hughes et al. [41], who has described diet as one of the most important factors that influence group uniformity.

The animals used for this study were 30-days old chickens from medium-growth lines, which after the first month did not show a complete productive development. This is the reason for some differences observed when the results of this study were compared with those reported in previous studies that usually use fast-growing strains. This is due to the degree of maturity of the chicks depending on the breed studied [42].

Although the slaughter weight (W5) increased, not differences were found in the weight of the front quarters and the breast. These results are consistent with the majority of studies on broilers and quail, where despite having increased the final weight of the animal, the weight of the breast, and the composition of the different parts of the carcass in general (wings, thighs, and breast mainly), were not affected by the inclusion of *Musca domestica* (MD) or *Hermetia illucens* (HI) in the chicken diets [28,30,43,44,45]. On other hand, Hwangbo et al. [38] and Pretorius [46] found significantly higher breast weights in those animals that received insect meal compared to the control. Hwangbo et al. [38] attributes the differences between the treatment and control groups with respect to breast weight to a higher rate of protein accumulation with the inclusion of MD, due to the optimal profile of essential amino acids of the latter (particularly lysine) and a high digestibility of protein, although the lack of concordance between the consulted studies can be attributed to the different nutritional composition of the insect species used in the studies and with the variation on the composition [46]. The results clearly indicate that the inclusion of insects in the diet of chickens has the potential to be food sources, which produces carcasses and their portions (breasts, wings and thighs) of similar size compared to those in which chickens received diets containing traditional feeding ingredients [46].

No differences were found in nutritional breast composition. According to our results, other authors [13,28,30,43,47] indicated the absence of effects of the inclusion of insect meal in the diet on the chemical composition of the breast. In contrast Schiavone et al. [48] published that the increase in HI in the chicken diet caused a drop in humidity and increased protein. On the other hand, Ballitoc and Sun [34] showed an increase in moisture and protein in the breast of the group that received 2% TM. However, the explanation for this situation is not entirely clear and further investigation must be carried out. The results also showed that an increasing replacement of the feeding with CLM did not affect the oxidative state of the meat since the chicken breasts meat showed similar TBARS values in the four groups. These results correlate with those obtained by Cullere et al. [6] who did not found variations in oxidative state when insect larval meal was included in the quail diet.

Then, the observed results suggest that the composition of the chicken breast meat (in terms of moisture, ash and fat) and the oxidative state of chickens fed with insects, is comparable with birds which were fed conventional diets. This is a key aspect from the nutritional point of view to favor the commercialization of this type of products. Probably, the apparent differences in the diet (in terms of protein), had an impact on the productive development of the animals, but without modifying the chemical composition of the carcass. This promising result reinforces the potential of this innovative ingredient as a poultry feeding.

Regarding meat fatty acid composition, the incorporation of CLM, caused a gradual increase in the content of palmitoleic acid that was directly correlated with the percentage of CLM inclusion in the chicken diet. This result is in agreement with the composition of the feed (Table 3) and it is related with the individual composition of CLM fatty acids (Table 1), where palmitoleic acid was the third acid with the highest concentration. Likewise, despite the differences were not significant, an increase in the concentration of oleic acid and a drop in the concentration of stearic acid (C18:0) was observed in chicken breast meat of groups that received higher inclusion of CLM in the diet (T3 and T4) with respect to the control (T1) and T2 group. These results are in agreement with what is observed in the fatty acid composition of dietary treatments. It was observed that the main fatty acid in the CLM composition was oleic acid (Table 1), in this way as the concentration of CLM in the feed increased, the amount of this acid in the different treatments increased (T2, T3 and T4). Likewise, the lower content of stearic acid observed in the feeds that contained a higher concentration of CLM (Table 3) is reflected in a lower concentration of the acid in the chicken breast meat.

Indeed, it was observed that chickens fed with CLM in diet (T2, T3 and T4), had a significant lower content of α-Linolenic acid (ALA C18: 3 n-3) and also a lower but not significant content of ɣ-Linolenic acid (GLA C18: 3 n-6) in the chicken breast meat (Table 6). These results are in agreement with the observed in the individual composition in fatty acids of the experimental feeds (Table 3). Likewise, a not significant increase of myristic acid level (C14:0) was observed, as the replacement of the control feed with CLM does, which is related to the content of this acid in the experimental diets (Table 3). These results are in agreement with that described by by Loponte et al. [49], who found similar results, relating this fact to a higher concentration of myristic acid in insects and that could subsequently affect the composition of the chicken breast meat. Similar, Cullere et al. [13] described that the dietary inclusion of HI greatly changed the proportions of fatty acids in quail breast meat (increasing the concentrations of C10:0, C12:0, C14:0, C16:0 and C20:0). Other authors [46,48] described an increase in the content of lauric, myristic and palmitic acid (C12:0, C14:0, C16:0) in chicken breast meat, when insect meal was included in the diet. Dabbou et al. [28] showed that the group that included TM showed higher percentages of oleic and ALA, a trend towards higher MUFA rates and lower palmitic and saturated fatty acid (SFA) rates at the same time. Dabbou et al. [28] related this fact to the fatty acid composition of the TM species, oleic acid being the predominant one in the larval form. Dabbou et al. [28] described that chicken breast meat lipids are mainly composed of triacylglycerol and phospholipids, the latter being rich in very long chain n-3, mainly eicosapentaenoic acid (EPA; C20:5 n-3) and acid docosahexaenoic (DHA; C22:6 n-3), which are well known for their high biological efficacy in the body and their beneficial effects on human health. While our results did not show variations in the content of DHA and EPA [6,28] found a significant reduction in DHA content in poultry breast meat fed with increasing levels of HI.

A significant increase in content of SFA was observed for T2 group when it was compared with T3 group. This fact would require new studies to verify this result. On the other hand, our results for the content of SFA, n-3, n-6 were slightly higher than those obtained by Loponte et al. [49] and lower for content in MUFA and lower for content in MUFA. These authors did not found variations in the content of the main groups of fatty acids (SFA, MUFA and PUFA) in chicken breast meat of the different dietary treatments that included TM versus the control as observed in this study. These authors considered that, despite the great differences found in terms of the fatty acid profile of the TM composition compared to soybean meal, (particularly in the content of SFA and n-6), no variations in the composition were observed. Otherwise, study published by Cullere et al. [13] showed an increase in content of SFA and MUFA, which increased from the control treatment to the different percentages of inclusion of HI larval meal. In contrast, these authors show PUFA level decreased significantly from the control treatment to those that included larval meal in the diet, with the n-3 fraction showing the greatest decrease. Ref. [47] observed that supplementation of HI oil in the diet of broilers increased SFA and decreased PUFA in breast muscle, but did not affect MUFA content. Schiavone et al. [48] published that the MUFA increase due to the high oleic content observed as a result of the inclusion of HI flour levels in the chicken diet.

Atherogenic (AI) and thrombogenic (TI) index correlate the different amounts of some specific SFA, MUFA and PUFA of the n-3 and n-6 series. They have been proposed to indicate the contribution of these fatty acids to the prevention or promotion of pathological phenomena in humans, such as atheromas and/or the formation of thrombi [26]. The results showed an increase (*p* < 0.05) for the TI value in group T2 with respect to T1; while the AI was not affected. It should be noted that, in both groups, AI and IT values were low, and could be considered healthy for consumers [50,51], since recommended AI values are below 0.5. These results are in agreement with those reported by Loponte et al. [49]; who did not observe any difference between the breast meat of broilers fed with larva TM meal and those fed with soy in terms of quality (n-6/n-3 ratio, AI and TI). On the other hand, Dabbou et al. [40] showed that the TM group had significantly lower AI and TI in chicken breast meat compared to the control group.

The lack of agreement between the different studies on the composition of the meat when insects are included in the diet, could be related not only with the typical lipid profile of the species of insect used but also determined by the insects breeding substrate. In fact, as already mentioned above, it has been shown that the ether extract content of the larvae can vary greatly depending on the substrate, as well as the fatty acid profile [7].

The L* values obtained in this study were similar to those shown by previous authors when they studied medium-slow growing strains [52,53]. The values obtained for a* and b* parameters are slightly higher than those obtained for other breasts of the same lineage (RedBro) slaughtered after 120 days when they have reached their full development [40]. It can be due to the precocity and lack of maturity of the chickens selected for this study (30 days old). No significant differences were observed for L* parameter depending on the diet received. This coincides with what is described by other authors [9,28,43] who did not found variations for L* when larval meal was included in the diet. Likewise, Bovera et al. [30] showed that this parameter was not modified in the breast of the animals that had been fed with TM, neither on the raw breast, nor cooked. These authors affirm that the absence of differences would allow this meat to be perfectly accepted by consumers. Similarly, for the parameters a* and b* no significant differences were observed between the different treatment groups. These results are in agreement with those described by previous authors [13,28,38,43] between the different groups. Although there were no significant differences, the a* value showed a numerical reduction when CLM was included in the diet, especially in the T4 group. Similar results were those shown by Pretorius [46] who found significantly lower values for a* and b*, while Pieterse [43] only found a reduction in the a* value in animals that had received insects in their diet. In contrast, Schiavone et al. [48] showed a significant increase in the redness of the meat (a*) as the concentration of HI in the feed increased related to a possible accumulation of pigments from the insect meal in the intramuscular fat. These authors also reported a linear decrease in the b* value when the HI concentration was increased, attributing it to a progressive decrease in the content of corn in the diet, more than to the effect of the inclusion of insects in the diet.

No differences for the pH value were observed between the different treatments. Previous studies [6,46,48] did not find differences for the pH value as a function of the inclusion of insect flours in the feed. However, Cullere et al. [13] showed lower pH values in the chicken breast meat when larval meal was included in the chicken diet, while Bovera et al. [30] found a significant increase in pH in the meat of birds that had incorporated TM in the diet. These differences found between the different studies could be conditioned by the glycogen content in the muscle at the time of slaughter, which is directly related to the stress suffered prior to slaughter. Other authors, [40] attributed these differences to the rearing system and the genotype used in the different studies.

Cooking losses were higher (*p* < 0.001) in the control group (T1) than in the groups that included CLM in the diet (T2, T3, T4). It should be noted that the control group (T1) presented slightly higher humidity values, although without differences with the rest of the treatments, and a pH slightly lower than T1 and T2. This would suggest that the meat from animals that have received CLM in the diet would have a better aptitude for the conservation and processing of the meat as the moisture content and cooking losses are reduced. On the other hand, Schiavone et al. [48] did not found differences between the treatments while the studies carried out by Bovera et al. [30], and Cullere et al. [44] showed higher values of cooking losses in those groups that had included a higher concentration of insect in the bird’s diet. This was justified based on the fact that meat with a pH close to the protein isoelectric value (5.2 to 5.5) resulted in a lower water holding capacity, which produces a more intense loss of cooking.

It was observed that the pH did not affect the water holding capacity (WHC), measured through the percent of expressible juice which did not showed significant differences between the treatments. This is in agreement with [46] that also found no differences between the different groups for drip losses. An increase of Warner Braztle Shear Force value was observed as the inclusion of CLM in the feeding increased; however, the differences were not statistically significant. This is in agreement with Bovera et al. [30] that did not find significant differences between the groups that included TM in the diet, although as in the present study, WBSF was higher in the chicken breast meat of the chickens fed with TM. In this way, it could be argued that the increase in resistance, together with the decrease in cooking losses, could be related to some structural change in the disposition of the proteins as a consequence of the inclusion of these alternative sources. However, more in-depth studies are required to assess whether or not there are modifications to the conformation and protein composition derived from the inclusion of CLM. On the other hand, Cullere et al. [13] described softer meats when the broilers quails had consumed insect meal in their diet.

The results show the ability of the method to discriminate and to successfully differentiate samples from the control group (T1). This fact is reflected in the Figure 1 where the average spectrum of control group appears separated from the rest of the spectra. Regarding the different treated groups, the ability of the method to correctly discriminate increased from group T2 to group T4. It was obtained that the average success rate of the method was 92.1%. These findings are in agreement with those found by Zamora-Rojas et al. [54] who described that the NIR system was able to classify more than 90% of pig carcasses correctly based on the feed received. Ripoll et al. [55] described that NIR spectroscopy was able to successfully classify lamb meat from three different feeding systems. Similar results were found by Berzaghi et al. [56] who showed that the performance of the discriminant models had a correct classification of 100% between the chickens that had received the control diet and those that had received the enriched diets. The results derived from this study suggest that, due to its speed of analysis and low operating costs, the NIRS system can be used as a helpful tool to discriminate poultry meat from different dietary treatments.

## 5. Conclusions

*Calliphora* sp. Larva meal can provide sufficient nutrients (in terms of the chemical composition studied) for the feeding of chickens. In addition, they contain sufficient amounts of aminoacids, fatty acids and other nutrients necessary for raising chickens in organic systems. It was observed that the weight of the chickens that had received a greater amount of CLM (T3 y T4) in the diet had a greater final body weight. The group with the highest inclusion of CLM (T4) showed a greater total ADG for the study period and a lower FCR. However, no proportionality was observed in the improvement on the productive parameters with respect to the concentration of CLM included in the feed (T2, T3, T4). This study suggests that the partial substitution of the compound feed formulated with soybean meal for CLM could be suitable in the feeding of chickens during the initial phase of life.

T4 chicken’s meat showed a lower value of cooking losses and a higher content of palmitoleic acid. The rest of the quality parameters studied were not affected by the inclusion of CLM in the feed. Therefore, it can be concluded that the partial replacement with *Calliphora* sp. in the starter diet of medium-slow growing chickens reared in organic systems (up to 15% inclusion level) is technically feasible and provides meat of a quality that is broadly comparable to that of chicks fed a conventional diet.

The NIRS correctly classified 92.1% of the samples based on the diet received, which makes this system a potentially useful instrument to differentiate meat based on the diet received.

## Figures and Tables

**Table 1:** Nutritive value and fatty acid composition of *Calliphora* sp.

**Nutrient**	**g/100 g DM**
Moisture	8.10
Crude Fiber	12.30
Crude Fat	26.60
Crude protein	48.50
Ash	4.50
**Fatty acid profile**	**g/100 g of fat**
Lauric A. (C12:0)	0.14
Myristic A (C14:0)	2.90
Myristoleic A. (C14:1)	0.42
Pentadecanoic A (C15:0)	0.03
Pentadecanoic A.(C15:1c)	0.04
Palmitic A. (C16:0)	29.28
Palmitoleic A. (C16:1)	17.72
Margaric A. (C17:0)	0.23
Margaroleic A. (C17:1 n-10)	0.28
Stearic A. (C18:0)	3.12
Oleic A. (C18:1 n-9c)	38.05
Trans-elaidic A (C18:1 n-9t)	0.05
Linoleic A. (C18:2 n-6c)	6.22
α-linolenic A. (C18:3 n-3)	0.19
γ-linolenic A (C18:3 n-6)	0.02
Arachidic A.(C20:0)	0.07
Eicosenoic A. (C20:1 n-9)	0.15
Arachidonic A. (20:4 n-6)	0.17
Eicosapentaenoic A. (C20:5 n-3)	0.37
Docosadienoic A. (C22:0)	0.05
Docosatretanoic A. (C22:4 n-6)	0.20
SFA	35.83
MUFA	56.61
PUFA	7.57
SFA/ΣPUFA	4.73
n-3	0.56
n-6	0.39
n-6/n-3	0.69

**Table 2:** Ingredient and nutrient content of the different dietary treatment.

Treatment	T1	T2	T3	T4
**Ingredients (g/100 g)**				
Larva *Calliphora* sp.	0	5	10	15
Soybean meal	35.19	33.43	31.67	29.91
Corn	30.00	28.50	27.00	25.50
Wheat	12.87	12.23	11.58	10.94
Barley	9.84	9.35	8.86	8.36
Spring peas	8.00	7.60	7.20	6.80
Bicalcium Phosphate	1.93	1.83	1.74	1.64
Calcium carbonate	0.82	0.78	0.74	0.70
Premix ^1^	0.50	0.48	0.45	0.43
Acidifier	0.30	0.29	0.27	0.26
Common Salt	0.28	0.27	0.25	0.24
Sodium bicarbonate	0.16	0.15	0.14	0.14
Enzymatic complex	0.10	0.10	0.09	0.09
Total	100.0	100.0	100.0	100.0
**Chemical composition (g/1000 g)**				
Digestible Energy (kcal)	2699	2858	3016	3175
Moisture (105 °C)	9.24	9.35	9.19	8.92
Crude protein	21.45	24.09	25.49	25.98
Crude Fiber	3.68	4.15	3.51	3.21
Crude Fat	5.69	7.10	8.25	8.40
Ash (550 °C)	7.09	6.81	6.76	6.66
Starch	38.48	35.87	33.70	32.28
Calcium	0.86	1.47	2.08	1.47
Total Phosphorus	0.84	0.80	0.75	0.80
Methionine	0.33	0.07	0.12	0.16
M + C	0.70	0.67	0.63	0.60
Lysine	1.22	0.70	0.76	0.82
Tryptophan	0.27	0.26	0.25	0.23
Threonine	0.83	0.50	0.55	0.59
Arginine	1.51	0.79	0.85	0.90
Valine	1.04	0.40	0.44	0.49
Isoleucine	0.95	0.28	0.31	0.33
Salt	0.35	0.34	0.32	0.30
Sodium	0.17	0.16	0.15	0.14
Potassium	0.99	0.94	0.89	0.84
Chlorine	0.22	0.21	0.20	0.19

^1^ Organic Premix (Nutega Coslada, Madrid) Values given (g) per kg of feed: 1.23 Calcium; Dry matter 4.87; Values given (mg) per kg of feed. E5 Manganese (manganese oxide): mg/kg 65.0; E6 Zinc (zinc oxide) 37.0; E4 Copper (cupric sulfate pentahydrate) 4.0; 3b202 Anhydrous calcium iodate: 1.90; E8 Selenium (sodium selenite) 0.10; E1 Iron (ferrous carbonate) 18.0; 3rd 711 Vitamin K3 1.50; Vitamin B2 3.00; 3115 Niacinamide 15.0; 3a841 Calcium D-pantothenate 6.44; 3a890 Choline chloride 245.00. Values given (IU) per kg of feed 3a672a Vitamin at 7500.00; E671 Vitamin D3 150,000; Vitamin B12 (mcg/kg) 10.

**Table 3:** Fatty acid profile of experimental diets (g per 100 g of fat).

	T1	T2	T3	T4
Diet Composition (g/100 g)				
Lauric A. (C12:0)	0.12	0.12	0.12	0.12
Myristic A. (C14:0)	0.34	0.47	0.60	0.73
Myristoleic A. (C14:1)	1.73	1.67	1.60	1.54
Pentadecanoic A. (C15:0)	0.12	0.11	0.11	0.10
Pentadecanoic A.(C15:1)	0.00	0.00	0.00	0.01
Palmitic A. (C16:0)	38.26	37.81	37.36	36.91
Palmitoleic A. (C16:1)	0.47	1.36	2.19	3.05
Margaric A. (C17:0)	0.61	0.60	0.58	0.56
Margaroleic A. (C17:1 n-10)	0.09	0.10	0.11	0.12
Stearic A. (C18:0)	12.34	11.88	11.42	10.95
Oleic A. (C18:1 n-9c)	35.49	35.62	35.75	35.88
Trans-elaidic A. (C18:1 n-9t)	0.12	0.12	0.11	0.11
Linoleic A. (C18:2 n-6c)	6.66	6.64	6.61	6.59
α-linolenic A. (C18:3 n-3)	0.25	0.25	0.25	0.24
γ-linolenic A. (C18:3 n-6)	1.38	1.31	1.24	1.17
Arachidic A.(C20:0)	1.38	1.31	1.25	1.18
Eicosenoic A. (C20:1 n-9)	0.07	0.07	0.07	0.08
Arachidonic A. (20:4 n-6)	0.10	0.10	0.10	0.11
Eicosapentaenoic A. (C20:5 n-3)	nd	0.02	0.04	0.06
Docosadienoic A. (C22:2 n-6)	nd	0.00	0.01	0.01
Docosatretanoic A. (C22:4 n-6)	0.20	0.20	0.20	0.20
SFA	55.89	54.88	53.88	52.88
MUFA	37.91	38.85	39.78	40.72
PUFA	6.20	6.27	6.34	6.41
SFA/PUFA	9.01	8.80	8.58	8.37
n-3	0.25	0.27	0.28	0.30
n-6	0.36	0.36	0.36	0.37
n-6/n-3	1.44	1.41	1.37	1.33

nd: not detected.

**Table 4:** The effect of experimental diets on the growth performance of chickens.

	T1	T2	T3	T4	*p*
	**(g)**				
W1	0.049 ± 0.001	0.047 ± 0.001	0.050 ± 0.000	0.049 ± 0.001	0.284
W2	0.130 ^bc^ ± 0.003	0.126 ^c^ ± 0.004	0.147 ^a^ ± 0.010	0.145 ^ab^ ± 0.003	0.002
W3	0.281 ^ab^ ± 0.006	0.308 ^a^ ± 0.012	0.263 ^b^ ± 0.010	0.315 ^a^ ± 0.012	0.003
W4	0.525 ^b^ ± 0.015	0.523 ^b^ ± 0.017	0.585 ^ab^ ± 0.020	0.589 ^a^ ± 0.016	0.005
W5	0.787 ^b^ ± 0.022	0.751 ^b^ ± 0.021	0.868 ^a^ ± 0.020	0.874 ^a^ ± 0.026	0.001
	**(g/day)**				
ADG2-1	0.011 ^b^ ± 0	0.011 ^b^ ± 0.001	0.014 ^a^ ± 0.001	0.014 ^a^ ± 0.001	0.002
ADG3-2	0.022 ^ab^ ± 0.001	0.026 ^a^ ± 0.002	0.017 ^b^ ± 0.001	0.024 ^a^ ± 0.002	0.001
ADG4-3	0.035 ^bc^ ± 0.001	0.031 ^c^ ± 0.002	0.046 ^a^ ± 0.001	0.039 ^ab^ ± 0.001	0.001
ADG5-4	0.037 ^b^ ± 0.002	0.033 ^b^ ± 0.002	0.040 ^a^ ± 0.001	0.041 ^a^ ± 0.002	0.017
ADGt	0.026 ^bc^ ± 0.001	0.025 ^c^ ± 0.001	0.029 ^ab^ ± 0.001	0.029 ^a^ ± 0.001	0.001
Feed Intake (g)	1.165 ^a^ ± 0.025	1.076 ^c^ ± 0.002	1.098 ^b^ ± 0.001	1.080 ^c^ ± 0.002	0.001
FCR	1.600 ^a^ ± 0.051	1.547 ^a^ ± 0.043	1.357 ^b^ ± 0.04	1.331 ^b^ ± 0.405	0.001
	**Consumption per animal and study period of these nutrients (g)**				
Crude Protein	0.250 ± 0.008	0.259 ± 0.040	0.280 ± 0.050	0.281 ± 0.045	
Methionine	0.004 ± 0.002	0.001 ± 0.000	0.004 ± 0.001	0.004 ± 0.001	
Lysine	0.014 ± 0.000	0.008 ± 0.001	0.008 ± 0.001	0.009 ± 0.001	
Arginine	0.018 ± 0.001	0.009 ± 0.001	0.009 ± 0.002	0.010 ± 0.002	
Digestible Energy (kcal)	3144.335 ± 105.540	3075.208 ± 480.358	3311.568 ± 586.060	3429.000 ± 549.697	

Results are presented as mean ± standard deviation. ^a–c^: different superscripts indicate significant differences within a row (*p* < 0.05).

**Table 5:** Effect of the inclusion level of CLM and the slaughter on the weight of the quarter and breast, and the composition characteristics in the chicken breast meat (m. *Pectoralis major*) of the different treatments.

Meat Quality	T1	T2	T3	T4	*p*
Front quarter (kg)	0.11 ± 0.02	0.1 ± 0.01	0.12 ± 0.01	0.11 ± 0.01	0.131
Breast (kg)	0.07 ± 0.02	0.05 ± 0.02	0.07 ± 0.02	0.07 ± 0.01	0.104
Moisture (%)	75.84 ± 1.33	75.04 ± 0.86	74.94 ± 4.37	75.18 ± 2.71	0.851
Ash (%)	1.15 ± 0.04	1.15 ± 0.09	1.20 ± 0.16	1.20 ± 0.10	0.567
Fat (%)	1.37 ± 0.81	1.04 ± 0.56	1.67 ± 1.41	1.19 ± 0.81	0.388
TBARS (mg MDA/kg of meat)	0.057 ± 0.01	0.054 ± 0.00	0.054 ± 0.01	0.056 ± 0.006	0.521

Weight of the quarter and the breast are in kg. For moisture. ash. and total fat the values are shown in percent. For TBARS the value is shown as mg MDA/kg of meat. Results are presented as mean ± standard deviation.

**Table 6:** Fatty acid profile of chicken breast meat (g/100 g of fat) for each of the treatments studied.

Fatty Acid Profile	T1	T2	T3	T4	*p*
Lauric A. (C12:0)	0.50 ± 1.07	0.29 ± 0.25	0.17 ± 0.23	0.14 ± 0.07	0.538
Myristic A. (C14:0)	0.70 ± 0.35	1.6 ± 2.48	0.83 ± 0.56	0.91 ± 0.49	0.396
Myristoleic A. (C14:1)	0.10 ± 0.04	0.55 ± 0.39	0.22 ± 0.26	0.40 ± 0.46	0.220
Pentadecanoic A (C15:0)	0.12 ± 0.03	0.11 ± 0.01	0.11 ± 0.03	0.15 ± 0.06	0.086
Palmitic A. (C16:0)	21.13 ± 1.32	20.88 ± 1.33	20.02 ± 2.48	21.44 ± 0.95	0.172
Palmitoleic cis A. (C16:1 n-7c)	2.57 ^b^ ± 0.88	2.92 ^bc^ ± 0.85	3.94 ^ac^ ± 1.38	4.43 ^a^ ± 1.05	0.001
Margaric A. (C17:0)	0.21 ± 0.15	0.18 ± 0.01	0.17 ± 0.03	0.20 ± 0.03	0.336
Stearic A. (C18:0)	9.03 ± 2.33	9.81 ± 1.37	7.92 ± 2.10	8.96 ± 1.39	0.120
Oleic A. (C18:1 n-9c)	24.47 ± 6.04	24.16 ± 4.30	27.67 ± 5.68	25.8 ± 3.32	0.320
Trans-elaidic A. (C18:1 n-9t)	2.03 ± 0.43	2.28 ± 0.49	2.24 ± 0.75	2.33 ± 0.56	0.601
Linoleic A. (C18:2 n-6c)	27.95 ± 7.20	25.94 ± 3.41	27.3 ± 5.65	25.33 ± 3.12	0.598
α-linolenic A. (C18:3 n-3)	3.20 ± 0.98	2.43 ± 0.54	2.60 ± 0.94	2.33 ± 0.52	0.050
γ-linolenic A (C18:3 n-6)	0.36 ± 0.53	0.32 ± 0.29	0.20 ± 0.08	0.19 ± 0.05	0.204
Arachidic A.(C20:0)	0.12 ± 0.02	0.48 ± 0.75	0.15 ± 0.06	0.14 ± 0.05	0.384
Eicosadienoic A. (C20:2 n-6)	0.16 ± 0.10	0.34 ± 0.09	0.16 ± 0.14	0.20 ± 0.14	0.673
Eicosatetraenoic A. (C20:4 n-3)	0.74 ± 0.28	0.74 ± 0.28	0.56 ± 0.36	0.76 ± 1.07	0.368
Arachidonic A. (20:4 n-6)	4.26 ± 1.49	5.38 ± 1.66	4.36 ± 1.25	4.88 ± 1.69	0.081
Docosapentanoic A. (C22:5 n-3)	0.42 ± 0.21	0.35 ± 0.08	0.33 ± 0.21	0.40 ± 0.19	0.586
Eicosapentaenoic A. (C20:5 n-3)	0.40 ± 0.22	0.45 ± 0.35	0.31 ± 0.06	0.39 ± 0.33	0.332
Docosahexaenoic A. (C22:6 n-3)	0.28 ± 0.4	0.11 ± 0.11	0.21 ± 0.17	0.29 ± 0.01	0.838
SFA	31.80 ^ab^ ± 3.05	33.72 ^a^ ± 2.79	29.96 ^b^ ± 3.73	32.55 ^ab^ ± 1.95	0.030
MUFA	27.19 ± 5.79	27.53 ± 5.16	32.14 ± 4.94	31.16 ± 3.47	0.055
PUFA	40.47 ± 7.77	37.55 ± 4.33	37.69 ± 4.96	35.99 ± 3.77	0.258
SFA/ΣPUFA	0.83 ± 0.27	0.91 ± 0.16	0.81 ± 0.16	0.92 ± 0.13	0.399
n-3	3.75 ± 2.13	2.48 ± 0.96	2.82 ± 0.88	2.69 ± 0.74	0.110
n-6	36.33 ± 8.40	34.76 ± 4.50	34.55 ± 4.81	32.91 ± 3.42	0.533
n-6/n-3	11.84 ± 4.29	13.49 ± 4.29	13.62 ± 5.86	13.04 ± 3.83	0.789
AI	0.36 ± 0.03	0.41 ± 0.18	0.33 ± 0.06	0.38 ± 0.03	0.237
TI	0.56 ^ab^ ± 0.06	0.65 ^a^ ± 0.17	0.53 ^b^ ± 0.09	0.59 ^ab^ ± 0.05	0.035

Results are presented as mean ± standard deviation. ^a–c^: different superscripts indicate significant differences within a row (*p* < 0.05).

**Table 7:** Effect of the inclusion level of CLM on technological properties.

Technological Properties	T1	T2	T3	T4	*p*
L*	58.66 ± 3.43	56.00 ± 5.30	58.09 ± 2.15	57.55 ± 3.31	0.344
a*	8.23 ± 1.82	8.02 ± 2.09	8.14 ± 1.44	7.26 ± 1.20	0.452
b*	17.95 ± 1.42	16.24 ± 2.49	17.90 ± 2.31	16.31 ± 2.10	0.079
pH	5.54 ± 0.010	5.59 ± 0.11	5.59 ± 0.13	5.52 ± 0.13	0.247
Cooking losses (%)	17.06 ^a^ ± 5.97	10.79 ^b^ ± 4.22	11.38 ^b^ ± 5.20	8.60 ^b^ ± 2.00	0.001
Expressible juice (%)	12.74 ± 4.21	12.08 ± 3.87	13.11 ± 3.98	13.90 ± 4.89	0.747
Warner Bratzler Shear Force (WBSF) (N)	13.37 ± 2.43	14.25 ± 3.09	15.24 ± 4.91	16.72 ± 2.94	0.115

Results are presented as mean ± standard deviation. ^a,b^: different superscripts indicate significant differences within a row (*p* < 0.05).

## Data Availability

Data not available in public datasets, they can be consulted to the authors.
